# Life Stress and Complexity of Religious Identity

**DOI:** 10.1093/geroni/igaf122.3012

**Published:** 2025-12-31

**Authors:** Sophia Paleski, Alessia Petroni, Kyra Hultgren, Sarah MacDougall, Edward Thompson, Christopher Turner, Michiko Iwasaki, Andrew Futterman

**Affiliations:** Loyola University Maryland, Baltimore, Maryland, United States; Loyola University Maryland, Baltimore, Maryland, United States; Loyola University Maryland, Baltimore, Maryland, United States; Loyola University Maryland, Baltimore, Maryland, United States; College of the Holy Cross, Worcester, Massachusetts, United States; Loyola University Maryland, Baltimore, Maryland, United States; Loyola University Maryland, Baltimore, Maryland, United States; Loyola University Maryland, Baltimore, Maryland, United States

## Abstract

This study, using a random sample of 274 older adults and a “snowball” sample of 80 African American older adults (aged 57-100) living in Massachusetts, explores causes of having “straightforward” vs. “complex” orientation to religion in later life. Individual interviews included standard measures of religious affiliation, religious belief and behavior, religious doubts, physical and psychiatric health, life stresses and coping strategies, and social support networks. Personality characteristics were completed two times, 12 months apart, as a part of religious involvement and chronic stress in later life. Fifty-one individuals were identified in both samples with high levels of religious involvement and religious doubt – indicating cognitively complex, reflective religiousness (Batson, 1976). By contrast, 42 older adults were identified having extremely elevated levels of religious involvement with little if any religious doubt – indicating a more “straightforward” form of religiousness. Using a list of potential “causal” factors derived from interview data, discriminant function analyses were conducted to predict membership in either complex or straightforward religious groups. Initial analyses indicated that more education, being female, aged less than 80 years old, fewer functional impairments, onset of less severe, acute social stressors (e.g., breakup of relationships, loss of income) were associated with “complex” religiousness. By contrast, older age (> 80 yrs), increased infirmity, serious chronic stressors, and social isolation were associated with displaying more “straightforward” religiousness. These results are discussed considering our ongoing study of religious development using qualitative interview methods and previous research on cognitive and emotional complexity in later life (Labouvie-Vief, 2016).

